# Effects of norepinephrine on plaque hypoxia in atherosclerotic rabbits

**DOI:** 10.3389/fcvm.2023.1121413

**Published:** 2023-02-15

**Authors:** Jia-Yu Wang, Kun Liu, Yu-Bo Wang, You-Bin Deng, Jie Sun

**Affiliations:** ^1^Department of Medical Ultrasound, Tongji Hospital, Tongji Medical College, Huazhong University of Science and Technology, Wuhan, China; ^2^Department of Medical Ultrasound, The Third People’s Hospital of Hubei, Wuhan, China; ^3^Department of Medical Ultrasound, Maternal and Child Healthcare Hospital of Hubei, Wuhan, China

**Keywords:** atherosclerosis, norepinephrine, plaque vulnerability, hypoxia, contrast-enhanced ultrasound

## Abstract

**Background:**

Hypoxia plays a vital role throughout the whole process of atherosclerotic vulnerable plaque formation, which may be induced by a reduced oxygen supply. The vasa vasorum can be affected by norepinephrine (NE) and cause a reduced oxygen supply, ultimately leading to plaque hypoxia. This study aimed to investigate the effects of norepinephrine, which can increase the tension of the vasa vasorum, on plaque hypoxia, evaluated by contrast-enhanced ultrasound imaging.

**Methods:**

Atherosclerosis (AS) was induced in New Zealand white rabbits by a combination of a cholesterol-rich diet and aortic balloon dilation. After the atherosclerotic model was well established, NE was intravenously administered three times per day for 2 weeks. Contrast-enhanced ultrasound (CEUS) and immunohistochemistry staining were performed to evaluate the expression of hypoxia-inducible factor alpha (HIF-α) and vascular endothelial growth factor (VEGF) in atherosclerotic plaques.

**Results:**

The plaque blood flow decreased after long-term norepinephrine administration. The expression of HIF-α and VEGF in atherosclerotic plaques concentrated in the outer medial layers increased, which indicated that NE might cause plaque hypoxia by contraction of the vasa vasorum.

**Conclusion:**

Apparent hypoxia of atherosclerotic plaques after long-term NE administration was mainly caused by decreased plaque blood flow due to the contraction of the vasa vasorum and high blood pressure.

## 1. Introduction

Plaque rupture often leads to cardiovascular and cerebrovascular diseases. Therefore, evaluating vulnerable plaques and finding risk factors have been the focus of many studies. Studies have shown that hypoxia plays a vital role throughout the whole process of atherosclerotic vulnerable plaques formation and can promote the progression of plaques by various mechanisms (1, 2). Hypoxia deduced the ability of macrophages to excrete cholesterol, leading to the accumulation of lipids. In addition, hypoxia-inducible factor alpha (HIF-α), generated when hypoxia occurs, is closely involved in the inflammatory response of macrophages, leading to the production of cytokines like tumor necrosis factor-α and interleukin-6 as well as inducible nitric oxide synthase. Angiogenesis is a key feature of atherosclerosis. HIF-α mediates the expression of several genes, including endothelin-1, matrix metalloproteinase, and vascular endothelial growth factor (VEGF), which promote angiogenesis in plaques. Hypoxia and HIF-α also play an important role in vascular smooth muscle cell proliferation (3). Amount of hypoxic regions have been found within the vulnerable atherosclerotic plaques (4).

Hypoxia may be induced by a reduced oxygen supply due to an insufficient blood vessel network or defects in blood vessels. Neo-microvessels, the oxygen supply system of the plaques, are generated from the adventitial vasa vasorum and grow into the full thickness of the vessel wall (5, 6). As functional end arteries, the vasa vasorum is especially vulnerable to hypoxia (7). The status of the vasa vasorum determines the plaque blood volume and may influence the vulnerability of the plaque. The vasa vasorum has a regularly layered vascular structure of endothelial cells, vascular smooth muscle cells, and surrounding connective tissue, which is the same as other small-resistance arteries (8), can be affected by vasoconstrictor substances, such as norepinephrine (NE), and cause a reduced oxygen supply, ultimately leading to plaque hypoxia. The architecture of the vasa vasorum prevents the blood supply from reaching far enough from the adventitia into the media due to the pressure within the arterial wall (9). All these factors related to the vasa vasorum make plaques vulnerable to hypoxia. However, studies about hemodynamic changes in the vasa vasorum on plaque vulnerability and its mechanism are scarce (8). Contrast-enhanced ultrasound (CEUS) is a technique based on intravenous injection of an ultrasonographic contrast agent, specifically an intravenous contrast galactose microparticle suspension containing microbubbles with rheology similar to that of red blood cells (10). Previous research has determined that the enhanced intensity obtained using CEUS correlates well with tissue neovascularization (10, 11) and thus helps in evaluating the blood flow changes of atherosclerotic plaques (12, 13). Our previous research found that vasoactive agents can influence the degree of contrast enhancement of experimental atherosclerotic plaques during CEUS. Intravenous infusion of NE caused a decrease in the enhanced intensity in the atherosclerotic plaque, which indicated that the blood volume of atherosclerotic plaques could be decreased by vasoactive substances such as NE (14).

In this study, we used NE continuously to increase the tension of the vasa vasorum, and the changes in plaque blood volume after the increase in vasa vasorum tension were evaluated by contrast-enhanced ultrasound imaging. Pathological staining results reflecting plaque hypoxia were compared after the injection of NE. We attempted to determine whether the constant use of NE can cause plaque hypoxia.

## 2. Materials and methods

### 2.1. Experimental process

This study was approved by the Institutional Animal Care and Use Committee of Tongji Medical College, Huazhong University of Science and Technology. Atherosclerosis (AS) was induced in New Zealand white rabbits (male, average weight: 2.28 ± 0.19 kg). After a cholesterol-rich diet (consisting of 2% cholesterol, 3% egg yolk powder, and 3% lard) for 1 week, aortic balloon dilation was performed in all the rabbits, and then the cholesterol-rich diet was continued for 15 weeks. Standard ultrasound was performed to confirm the formation of atherosclerotic plaques and identify the pattern of each plaque. CEUS was performed to evaluate the baseline enhanced intensity (EI) of each plaque. Atherosclerotic rabbits were divided into an AS group and an AS + NE group. Rabbits in the AS + NE group were intravenously administered NE (1.5 μg/kg/min) three times per day for 2 weeks, while rabbits in the AS group were untreated. Then, standard ultrasound and CEUS imaging were repeated in the two groups, and the rabbits were sacrificed for HIF-α and VEGF staining.

### 2.2. Establishment of aortic atherosclerosis

Fifty New Zealand white rabbits approximately 2 months old with a weight of 1.8–2.5 kg (provided by Hubei Experimental Animal Center) were selected for the establishment of an atherosclerosis model. The rabbits were fed a high-fat diet consisting of 2% cholesterol + 3% egg yolk powder + 3% lard, 100 g in the morning and 50 g in the evening every day. Balloon dilation was performed after high-fat feeding for 1 week. The animals were anesthetized by injecting 1.5 ml/kg 3% pentobarbital sodium through the auricular vein. The right femoral artery was separated, the heparinized puncture needle was inserted parallel to the artery at a 20^°^ angle with the blood vessel, and then the balloon catheter with a diameter of 3.0–3.5 mm was pushed in. The catheter was connected to the lateral end of the pressure pump, and saline was injected. The pressure was maintained at 10–15 kPa. The balloon was dilated and slowly pulled out to the femoral artery bifurcation. After the operation, penicillin was intramuscularly injected at 80,000 units/d, and the high-fat diet was continued for 16 weeks.

### 2.3. Arterial blood pressure measurement

After anesthesia, the limbs and head of the rabbit were fixed, and the right median ear artery was tapped to fill it. After routine disinfection, 22G arteriovenous indwelling needle was used for puncture and catheterization, and the length of insertion was approximately 2–5 cm. The artery extension tube (tube filled with 0. 02% heparin saline) connected the invasive blood pressure transducer and to the manometer which had been set at 0. After the systolic and diastolic pressure and the heart rate were recorded, the indwelling needle was removed and the bleeding was stopped by compression.

### 2.4. Ultrasound imaging

#### 2.4.1. Routine two-dimensional ultrasound examination

**FIGURE 1:**
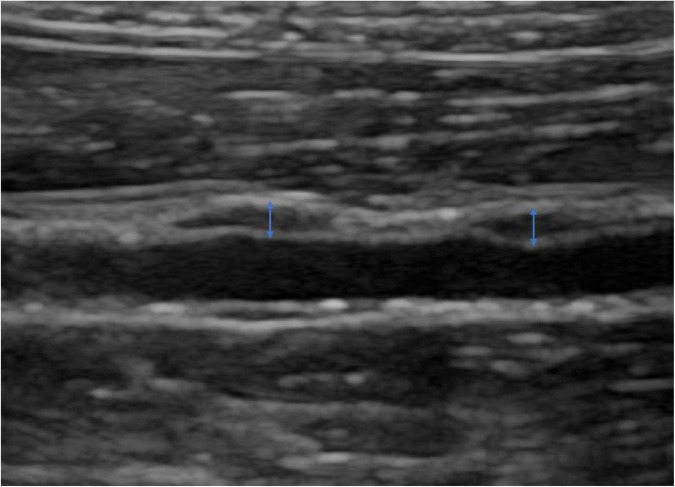
B-mode image of abdominal aortic plaque and arrows showed how to measure the thickness.

After anesthesia by intravenous injection of 3% pentobarbital (1.5 ml/kg), the experimental rabbit was placed on its left side on the examination table, and the right abdomen was disinfected and prepared. The Logiq E9 (GE Healthcare, Milwaukee, WI, USA) ultrasound instrument with a 9L probe at a transmission frequency of 12 MHz was used for routine two-dimensional ultrasound examination. Image depth was adjusted to 1–2 cm according to the depth of the plaque, and the focus position was set at the level of the plaque. The gain was adjusted to obtain a clear plaque image. The distance from the right renal artery, the thickness, and the echo type of each plaque were recorded (Figure 1).

#### 2.4.2. CEUS procedure

After the plaque was clearly shown in routine two-dimensional ultrasound examination, the contrast mode of the probe was switched, and image settings were adjusted to maximize visualization of the contrast signal. SonoVue (Bracco, Italy) was used as the contrast agent in this study. One milliliter of SonoVue was injected through the rabbit auricular vein within 1 s, and then 1 ml saline was injected into the indwelling needle. At the same time, the imaging procedure was started. The real-time and contrast-enhanced cine loop images from the appearance of contrast signals in the abdominal aorta to the elimination of the contrast were stored. After the CEUS procedure, NE was constantly injected at 1.5 μg/kg/min until the systolic blood pressure increased by 15 mmHg. This procedure was repeated every day for 2 weeks for each rabbit. Then, we repeated the CEUS imaging process of the selected plaque and sacrificed the rabbits by intravenous injection of excess pentobarbital.

### 2.5. Imaging analysis

**FIGURE 2:**
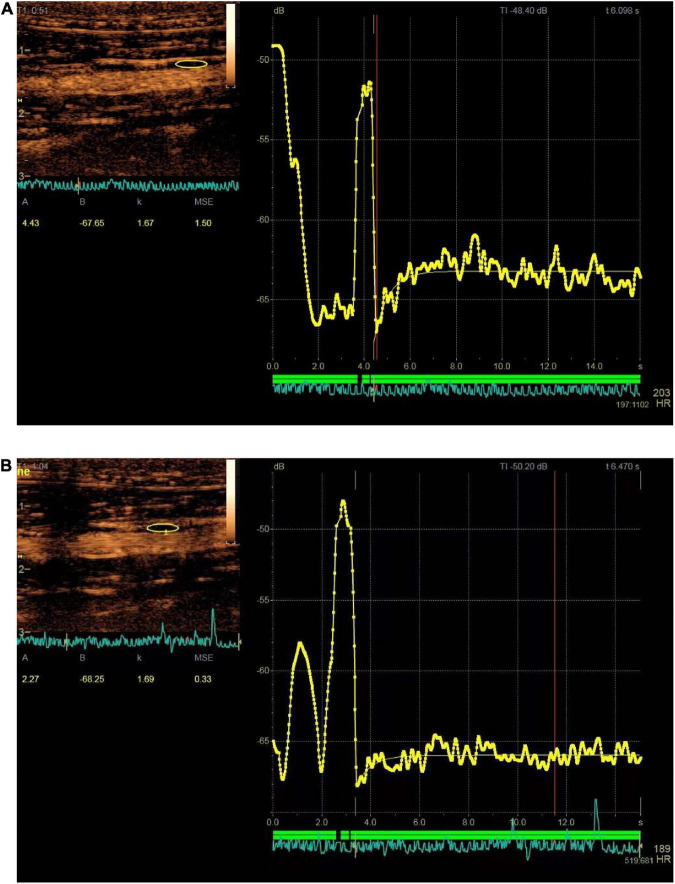
Time–signal intensity curves of plaque (yellow area and curve) at baseline and after administration of norepinephrine (NE) for 2 weeks. **(A)** Showed enhanced intensity before administration of NE (the enhanced intensity is 4.43 dB); **(B)** showed enhanced intensity after administration of NE for 2 weeks (the enhanced intensity is 2.27 dB).

The presence of enhanced spot echoes in the plaque during routine two-dimensional ultrasound examination is determined to be a calcified plaque, and vice versa for soft plaque. The portion of the plaque raised from the intima was measured as plaque thickness. Time–intensity quantitative analysis software (GE Healthcare, Milwaukee, WI, USA) was used to manually outline the edge of the plaque to draw the region of interest (ROI). The same shape of ROI was placed in the lumen as a reference for the plaque. The echo enhancement intensity (EI) of the plaque and lumen after CEUS injection can be automatically calculated (Figure 2).

### 2.6. Hematoxylin-eosin and immunohistochemistry staining

The plaques of atherosclerotic rabbits were soaked in 4% phosphate-buffered formalin (pH 7.4) for tissue fixation. Six micrometer sections of paraffin-embedded rabbit kidneys were stained with hematoxylin-eosin, anti-HIF-α antibody and anti-VEGF antibody. Image acquisition was performed on similar areas on the slices using a Nikon ELWD microscope equipped with a Nikon C-WB/22 camera.

### 2.7. Statistical analysis

SPSS software version 22.0 (SPSS, Chicago, IL, USA) was used for data analysis. Continuous variables are expressed as x ± s. Paired measurements at baseline and after intravenous infusion with NE were assessed by the paired t test. A *p*-value < 0.05 indicates statistically significant differences.

## 3. Results

### 3.1. Atherosclerotic model establishment

**FIGURE 3:**
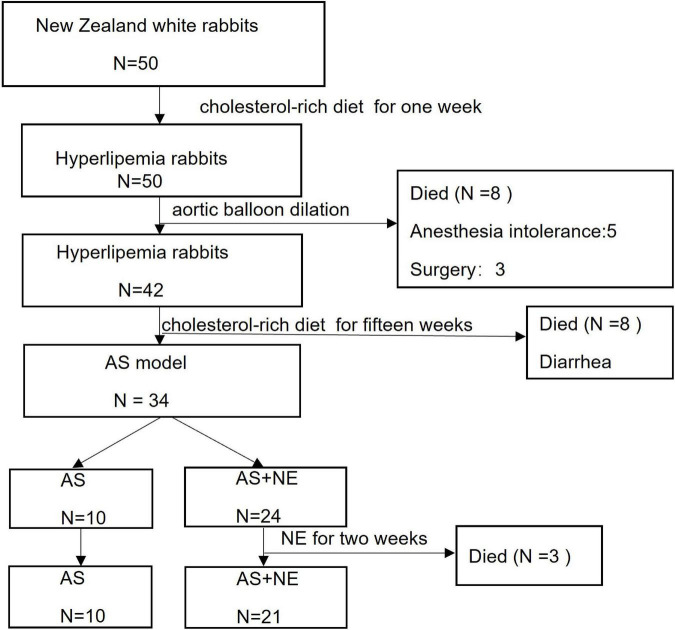
The number of deaths in this study. AS, atherosclerosis; NE, norepinephrine.

Fifty rabbits were enrolled in the study. Three rabbits died of anesthesia intolerance, five rabbits died on the second day after surgery, and eight rabbits died of diarrhea during a long-term cholesterol-rich diet. Three rabbits died during 2 weeks of NE administration. Finally, 31 atherosclerotic rabbit models were analyzed, including ten rabbits in the AS group and 21 rabbits in the AS + NE group (Figure 3).

### 3.2. Hemodynamic changes

After intravenous infusion with NE, the systemic blood pressure of the atherosclerotic rabbits increased by 29 ± 7% of its baseline level (*p* < 0.05), and the diastolic pressure increased by 25 ± 13% of its baseline level (*p* < 0.05). The heart rate decreased significantly (*p* < 0.05) during intravenous infusion with NE (Table 1). In contrast, systemic blood pressure, diastolic pressure, and HR in the control group showed no significant change (*p* > 0.05).

**TABLE 1:** Comparison of hemodynamic data at baseline and intravenous infusion of noradrenaline for the two groups.

	Baseline	2 weeks later	Change rate (%)	*t*	*P*
**AS + NE group (*N* = 21)**					
Systolic pressure (mmHg)	83.76 ± 9.268	107.9 ± 9.418	29.28 ± 6.732	25.98	<0.001
Diastolic pressure (mmHg)	60.81 ± 9.474	75.43 ± 10.21	24.97 ± 12.964	9.527	<0.001
HR (bpm)	253.1 ± 12.79	194.7 ± 35.48	23.19 ± 12.718	8.392	<0.001
**AS group (*N* = 10)**					
Systolic pressure (mmHg)	83.10 ± 9.53	79.40 ± 8.55	-3.38 ± 14.886	0.9518	0.3661
Diastolic pressure (mmHg)	65 ± 11.59	66.30 ± 7.89	3.12 ± 7.908	0.8351	0.4253
HR (bpm)	233 ± 17	238 ± 15	2.84 ± 10.539	0.6797	0.5138

### 3.3. Ultrasonography findings

**FIGURE 4:**
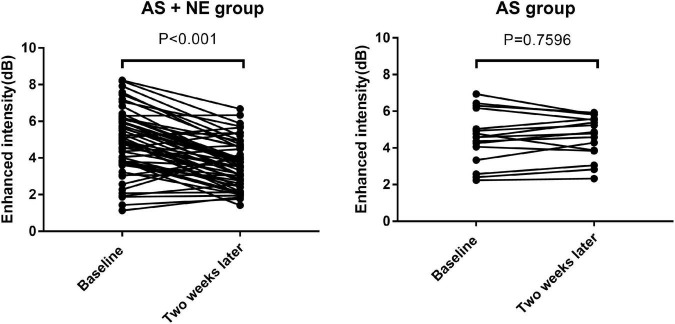
Enhanced intensity changes of plaques in the two groups. Vertical bars, mean value ± SD. AS, atherosclerosis; NE, norepinephrine.

**FIGURE 5:**
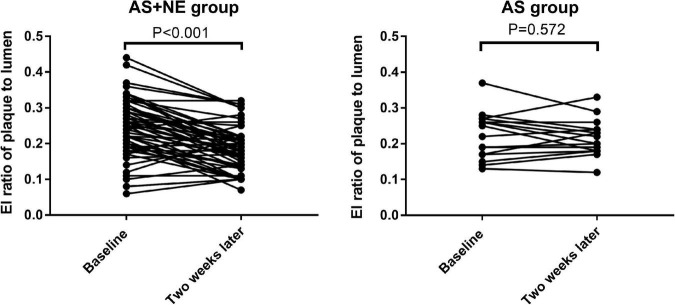
The changes in enhanced intensity ratio of plaque to lumen in the two groups. Vertical bars, mean value ± SD. AS, atherosclerosis; NE, norepinephrine; EI, enhanced intensity.

Seventy-one plaques were found in 31 atherosclerotic model rabbits, including 47 soft and eight calcific plaques in AS + NE rabbits and 14 soft and two calcific plaques in AS rabbits. The plaque thickness ranged from 0.60 to 1.20 mm (mean 0.77 ± 0.15 mm). There was no significant difference in plaques between the AS and AS + NE group at baseline (4.56 ± 1.44 vs. 4.90 ± 1.74 dB, *P* = 0.481). Two weeks later, EI in the AS group showed no significant change (4.56 ± 1.44 vs. 4.61 ± 1.15 dB, *P* = 0.760). The enhanced intensity of AS + NE plaques during CEUS imaging decreased from 4.9 ± 1.74 dB at baseline to 3.64 ± 1.27 dB after the last intravenous infusion of noradrenaline (*p* < 0.001) (Figure 4). The ratio of enhanced intensity of plaque to lumen decreased from 0.24 ± 0.08 to 0.18 ± 0.06 in the AS + NE group (*p* < 0.001), while it showed no significant difference in the AS group (0.22 ± 0.06 vs. 0.22 ± 0.05 dB, *P* = 0.717) (Figure 5).

### 3.4. Staining findings

**FIGURE 6:**
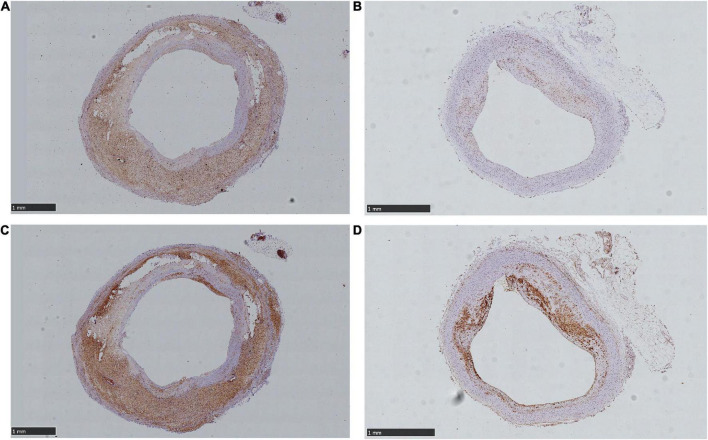
Different expression area of hypoxia- inducible factor alpha (HIF-α) and vascular endothelial growth factor (VEGF) between control and NE rabbit plaques. **(A)** Showed mount HIF-α expression of NE plaque. **(B)** Showed scattered and lower HIF-α expression of control plaque. **(C)** Showed mount VEGF expression of NE plaque. **(D)** Showed relatively little VEGF expression of control plaque. The arrows showed positive expressions.

The expression of HIF-α and VEGF showed high consistency in distribution and area among both the AS and AS + NE group. The AS group rabbits showed low expression of HIF-α and VEGF compared with the AS + NE group (6.972 ± 2.633 vs. 10.64 ± 3.234, 8.588 ± 2.476 11.75 ± 3.726), and the distribution was scattered (Figure 6). In contrast, after long term NE administration, AS + NE rabbits showed high HIF-α and VEGF expression, and the distribution was concentrated at the margins of the plaques (Figure 6 and Table 2).

**TABLE 2:** Different expression area of hypoxia-inducible factor alpha (HIF-α) and vascular endothelial growth factor (VEGF) between control and norepinephrine (NE) rabbit plaques.

	Control *N* = 16	NE *N* = 55	*t*	*P*
HIF-α (mm^2^)	6.972 ± 2.633	10.64 ± 3.234	4.149	<0.001
HIF-α/plaque area	0.3685 ± 0.07326	0.4509 ± 0.0877	3.42	0.0011
VEGF (mm^2^)	8.588 ± 2.476	11.75 ± 3.726	3.191	0.0021
VEGF/plaque area	0.4225 ± 0.08445	0.4849 ± 0.09363	2.394	0.0194

## 4. Discussion

Our study found that plaque blood flow decreased after long-term NE administration. The expression of HIF- α and VEGF in atherosclerotic plaques concentrated in the outer medial layers increased, which indicated that NE might cause plaque hypoxia by contraction of the vasa vasorum.

### 4.1. Vasa vasorum structure and neovascularization and plaque stability

The vasa vasorum consists of small arteries that enter the vascular wall either from major branches (vasa vasorum externa) or from the luminal surfaces (vasa vasorum interna) and then arborize to the outer media (15). A previous study showed that most of the neovessels in atherosclerosis sprout from the vasa vasorum in the adventitia, and only a small portion extend from the vessel lumen (16). As functional end arteries, the vasa vasorum exists mainly in the adventitial and outer medial layers of the blood vessels. The primary role of the vasa vasorum is providing oxygen and nutrients from the adventitia to the host vessel wall. Inner areas of the vessel wall are supplied by diffusion *via* the blood circulating in the lumen of the vessel (17, 18). When atherosclerosis occurs, the extension of the vasa vasorum to the full thickness of the media and intima of atherosclerotic plaques represents pathological neovascularization (8). Adventitial-derived vasa vasorum neovascularization is immature, lacks pericytes and is prone to leaking (19). The potentially noxious and inflammatory plasma components resulting from neovascularization increase plaque volume and lead to plaque rupture (6, 20).

### 4.2. Vasa vasorum hemodynamics

Unlike the neovessels within the plaque, the vasa vasorum possesses a relatively normal vascular structure, including the layered arrangement of endothelial cells, smooth muscle cells, and surrounding connective tissue. These structural characteristics make it prone to be affected by various vascular active substances in the body and control the blood supply to plaques (6). Infusion of NE was reported to induce a decrease in the blood flow of the vasa vasorum by directly constricting the vasa vasorum and raising the arterial blood pressure (21). An increase in the pressure in the arterial lumen or stent overexpansion could compress the vasa vasorum in the arterial wall and consequently decrease the density of the perfused vasa vasorum (8, 22). In our study, we found that constant administration of NE enhanced the intensity decrease, which indicated a decreased blood supply to plaques. As we illustrated before, the compression of the vasa vasorum induced by higher aortic pressure (increased by 29 ± 7% of its baseline level) and the shrinkage of the vasa vasorum induced by NE may account for this. Vasa vasorum can provide oxygen and nourishment to the outer third of the vascular media. The inner areas of the vessel wall are supplied by vasa vasorum interna, which obtains oxygen via the blood circulating in the lumen of the vessel. Therefore, the oxygen level is different in a plaque, and the outer third of the vascular media is prone to hypoxia, especially when the vasa vasorum contracts. In contrast to the lower and dispersive expression of VEGF and HIF in control rabbits, the expression in NE rabbits was higher and concentrated in the outer media of the plaques, which is consistent with the blood supply of the plaques illustrated above. The higher expression of HIF and VEGF makes plaques unstable by expressing proinflammatory cytokines and inducing angiogenesis by oxidative stress involving VEGF signaling (23), finally causing plaque rupture.

### 4.3. Contrast enhanced intensity and plaque hypoxia

Contrast agents used in CEUS are similar in size to red blood cells and do not leak out of blood vessels, making them ideal pool imaging agents. Therefore, the reflection intensity of the contrast agent during CEUS represents the blood volume of the plaque. Changes in contrast intensity indicate changes in blood volume. Many studies have shown that the density of neovascularization in plaques is related to the intensity of plaque enhancement during CEUS (24). In our study, we found that the intensity of plaque enhancement was decreased, and the ratio of plaque to lumen enhancement intensity was also decreased, indicating that plaque blood flow was decreased, which may be caused by the contraction of the plaque vasa vasorum affected by NE. Staining also proved that higher expression of HIF-α and VEGF appeared in the plaque, compared with the control group, indicating that plaque hypoxia may occur. However, the changes in the ratio of plaque enhancement intensity and luminal enhancement intensity may be related to the density of neovascularization in plaques, and the degree of the change showing hypoxia has not been studied thus far. More studies are needed to confirm this.

### 4.4. Limitation

Our study was relatively limited by the small sample sizes of plaques without the administration of NE. Further studies should be designed to explore the detailed mechanism of hypoxia.

## 5. Conclusion

After NE administration, the blood pressure in atherosclerotic rabbits was elevated, and the heart rates decreased. Plaque-enhanced intensity and its ratio to lumen were also reduced. Plaques showed massive expression of HIF-α and VEGF. These changes indicate that NE may reduce vasa vasorum blood flow and cause plaque hypoxia in atherosclerotic rabbits.

## Data availability statement

The raw data supporting the conclusions of this article will be made available by the authors, without undue reservation.

## Ethics statement

The animal study was reviewed and approved by Institutional Animal Care and Use Committee of Tongji Medical College, Huazhong University of Science and Technology.

## Author contributions

J-YW: methodology, statistical analysis, writing—original draft, and visualization. KL and Y-BW: investigation and formal analysis. Y-BD: resources and project administration. JS: conceptualization, methodology, writing—original draft, supervision, project administration, and funding acquisition. All authors contributing to the manuscript have approved the final version to be published.
